# JWES: a new pipeline for whole genome/exome sequence data processing, management, and gene‐variant discovery, annotation, prediction, and genotyping

**DOI:** 10.1002/2211-5463.13261

**Published:** 2021-08-11

**Authors:** Zeeshan Ahmed, Eduard Gibert Renart, Deepshikha Mishra, Saman Zeeshan

**Affiliations:** ^1^ Institute for Health, Health Care Policy and Aging Research Rutgers The State University of New Jersey New Brunswick NJ USA; ^2^ Department of Medicine Rutgers Robert Wood Johnson Medical School Rutgers Biomedical and Health Sciences New Brunswick NJ USA; ^3^ Rutgers Cancer Institute of New Jersey Rutgers The State University of New Jersey New Brunswick NJ USA

**Keywords:** gene, variants, database, whole genome, whole exome, bioinformatics application

## Abstract

Whole genome and exome sequencing (WGS/WES) are the most popular next‐generation sequencing (NGS) methodologies and are at present often used to detect rare and common genetic variants of clinical significance. We emphasize that automated sequence data processing, management, and visualization should be an indispensable component of modern WGS and WES data analysis for sequence assembly, variant detection (SNPs, SVs), imputation, and resolution of haplotypes. In this manuscript, we present a newly developed findable, accessible, interoperable, and reusable (FAIR) bioinformatics‐genomics pipeline Java based Whole Genome/Exome Sequence Data Processing Pipeline (JWES) for efficient variant discovery and interpretation, and big data modeling and visualization. JWES is a cross‐platform, user‐friendly, product line application, that entails three modules: (a) data processing, (b) storage, and (c) visualization. The data processing module performs a series of different tasks for variant calling, the data storage module efficiently manages high‐volume gene‐variant data, and the data visualization module supports variant data interpretation with Circos graphs. The performance of JWES was tested and validated in‐house with different experiments, using Microsoft Windows, macOS Big Sur, and UNIX operating systems. JWES is an open‐source and freely available pipeline, allowing scientists to take full advantage of all the computing resources available, without requiring much computer science knowledge. We have successfully applied JWES for processing, management, and gene‐variant discovery, annotation, prediction, and genotyping of WGS and WES data to analyze variable complex disorders. In summary, we report the performance of JWES with some reproducible case studies, using open access and in‐house generated, high‐quality datasets.

## Graphical abstract

In this manuscript, we present JWES, a newly developed FAIR bioinformatics‐genomics pipeline for gene‐variant discovery, annotation, prediction, and genotyping, and big data modelling and visualization. JWES is an open‐source and freely available application, allowing scientists to take full advantage of all the computing resources available, without requiring much computer science knowledge.

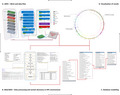

**Abbreviations**
API: application programming interface
BQSR: base quality score recalibration
BWA: Burrows–Wheeler Aligner
DNSNVs: de novo single‐nucleotide variants
FAIR: findable, accessible, interoperable, and reusable
ERD: entity relationship diagram
ETL: extraction, transfer, and loading
GEO: gene expression omnibus
GATK: genome analysis toolkit
hg38: human genome
HPC: high‐performance computing
I/O: input/output
JWES: Java based Whole Genome/Exome Sequence Data Processing Pipeline
ML: machine learning
NGS: next‐generation sequencing
OARC: Office of Advanced Research Computing
QC: quality check
SRA: sequence read architecture
SARS‐CoV‐2: severe acute respiratory syndrome coronavirus 2
SNPs: single nucleotide polymorphisms
VCF: variant call format
WES: whole‐exome sequencing
WGS: whole genome sequencing

Sequencing complex genomes has been a very challenging task [1]. Though, the sequenced genome analyses have revealed immense information about protein‐encoding transcripts, and single nucleotide polymorphisms (SNPs) [2]. There have been number of projects that set the firm foundation for the future biomedical research, including but not limited to Human Genome Project [3]. Data generated through the Human Genome Project not only revolutionized the research in field of human physiology, medicine, and development [4, 5], but fueled the future studies, including the ‘1000 Genomes Project’ specifically designed for the advancement in the understanding of genome variation among individual and populations [6]. Later, the 1000 Genomes Project was focused on the deeper characterization of genome variation for exploring the association between genotype and phenotype. The initial findings of this project described the precise location, allele frequency, and haplotype structure of around 15 million SNPs, 1 million short indels (insertions and deletions), and 20 000 structural variants [6]. These data further reinforced the next phase of study that provided a further extended list of 38 million SNPs, 1.4 million indels, and higher than 14 000 larger deletions. By using a mix of low‐coverage whole genome and exome sequencing approach, the 1000 Genomes Project established that individuals from different populations differ in their rare and common variants profile [7]. It provided a comprehensive knowledge of common genetic variation in the diverse set of individuals from different populations. Added approaches such as deep exome sequencing, microarray genotyping further helped in the completion of the project and led to a broader characterization of genetic variation, total exceeding 88 million variants, 3.6 million indels, and 60 000 structural variants [8]. The genome projects fulfilled the promise of genomics for medicine [9] by establishing implications of variant data analysis for the greater understanding of disease, by further community access to the data and multiple tools to help in diagnostics [10, 11, 12].

Technological advancements in genome sequencing in the recent past have revolutionized the field and made it more accessible and affordable [13]. The whole genome sequencing (WGS) [14] and the whole‐exome sequencing (WES) [15] are the most popular and widely adopted DNA sequencing technologies today. The WGS is applied to sequence the entirety of the genome and have broader coverage, while the WES is mainly used to only sequence the protein‐coding structures. These both sequencing techniques are extensively used to identify rare and common genetic variants in humans [16]. Along with the production of in‐depth and high‐quality DNA next‐generation sequencing (NGS) data [17], another challenge is to efficiently process raw WGS/WES data to support downstream analysis, interpretation, and visualization. Various bioinformatics tools have been developed worldwide to perform standalone and networked operations, which includes but not limited to, for example, cleansing of raw sequence data, converting raw signals into base calling, alignment with respect to the reference genome, identifying regions of interest in genome, assembly of contigs and scaffolds, and variant detection [18]. Despite immense technological progress, it is still a challenge among diagnostic laboratories and clinicians to timely find and interpret variants to unravel the genetic causes underlying diseases [15]. Genome Analysis Toolkit (GATK), maintained by the Broad Institute, has proven to be one of the widely adopted tool that offers different tools for variant discovery and genotyping [19]. Several bioinformatics‐genomics pipelines are freely available to process the WGS and WES data [20], which includes but not limited to the SeqMule [21], QIAGEN CLC Genomics [22], Galaxy [23], DNAp [24], STORMseq [25], ExScaliburn [26], Atlas2 [27], MC‐GenomeKey [28], Simplex [29], WEP [30], SeqBench [31], VDAP‐GUI [32], and fastq2vcf [33]. Most of the pipelines follow the similar workflow and are based on the nexus of different command‐line applications. Their execution not only requires good programming skills but deeper understanding of the computer science fundamentals, for example, UNIX commands, scripting and programming languages, high‐performance and/or cloud computing, database management, file structures, and formatting, etc. Furthermore, these pipelines do not support in efficiently managing high‐volume variant database modeling and visualization.

The WGS/WES approaches combined with RNA‐Seq can offer great sensitivity and serve as the gold standard for the precision medicine [34]. These techniques could hold the answers to the mysteries associated with varied drug response and disease infection rate in patients. However, one of the limiting factors associated is the incorporation of WGS/WES techniques in clinical practices [35]. There is a consistent increase in the numbers of individuals opting for elective genome sequencing, however not yet widely accessible [36]. Meanwhile, the need is to develop computing facilities with advanced technical infrastructure, and bioinformatics pipelines that can collect, process, analyze, and interpret the genomics data. We are focused on addressing these limitations [37]. In this manuscript, we present a newly developed findable, accessible, interoperable, and reusable (FAIR) bioinformatics‐genomics pipeline Java based Whole Genome/Exome Sequence Data Processing Pipeline (JWES) for variants discovery and interpretation, and big data modeling and visualization. The JWES is an open‐source and freely available pipeline, allowing scientists to take full advantage of all the computing resources available, without requiring much computer science knowledge.

## Methods

The JWES is a cross‐platform, hybrid, user‐friendly, and product line application that integrates multiple of the shelf open‐source command‐line tools. It is programmed in Java, designed to be deployed in a high‐performance computing (HPC) environment, and developed following software engineering principles for implementing efficient and user‐friendly bioinformatics applications [38]. In this manuscript, we are supporting the WGS and WES‐driven data processing, management, and gene‐variant discovery, annotation, prediction, and genotyping with the JWES. The overall workflow of the JWES consists of three different modules: data processing, storage, and visualization.

### JWES variant data‐processing

**Fig. 1:**
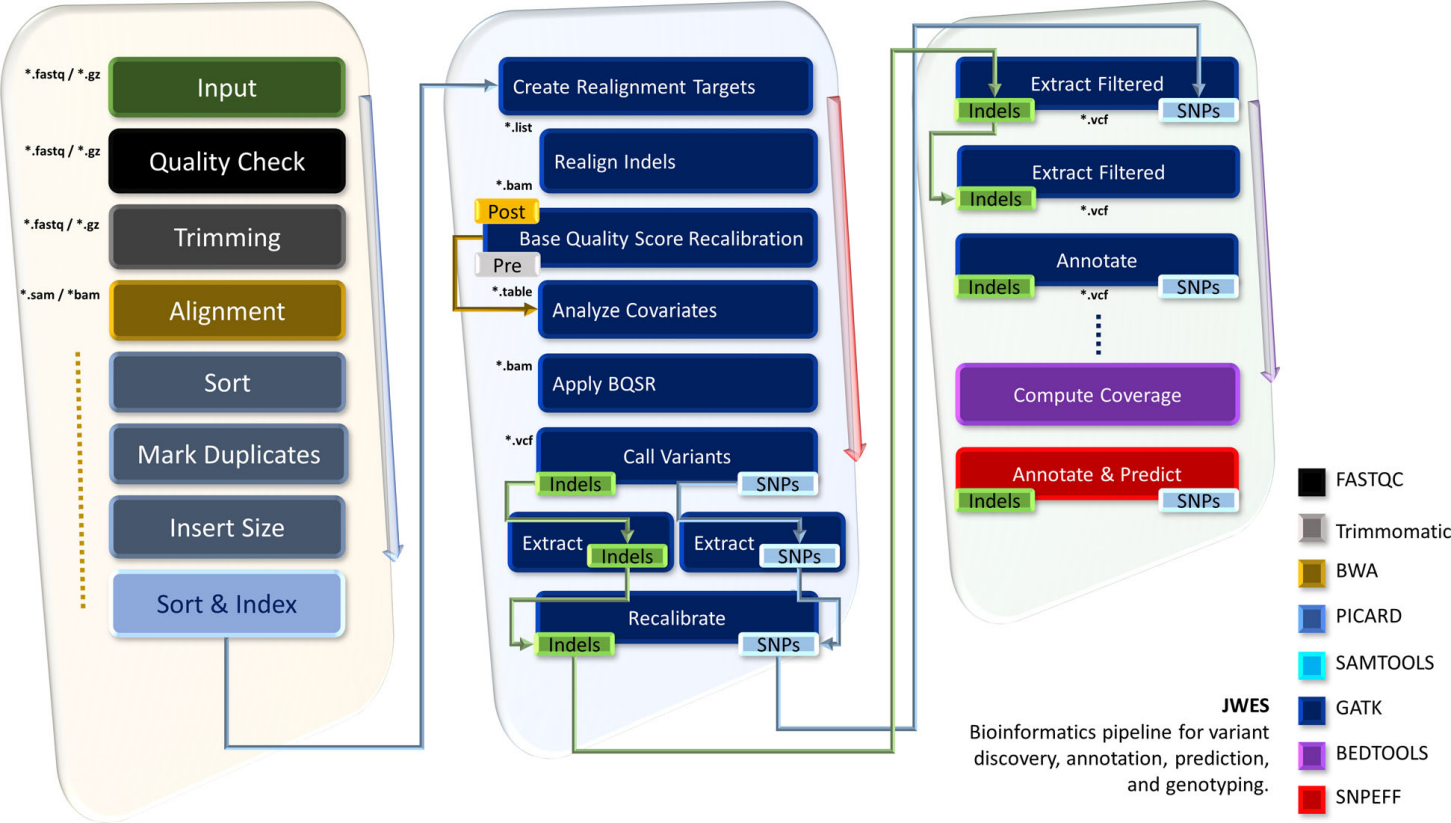
JWES pipeline for the whole genome and exome data processing, modeling, and downstream analysis. The figure explains all the data processing and analysis steps, which include input, QC, trimming, alignment, sort, mark duplicates, insert size, sort and index, create realignment targets, realign indels, Base Quality Score Recalibration (BQSR), analyze covariates, apply BQSR, recalibrate, extract filtered, compute coverage, annotate and predict.

The JWES data processing module is based on the concept of input/output (I/O) redirection (Fig. 1). It performs series of different tasks, which includes data quality check (QC); pruning barcodes and low‐quality sequences; indexing and alignment of sequences to the human reference genome; sorting and removal of duplicate sequences; and variant calling, extraction, annotation, and variant perdition. The JWES pipeline starts with the FastQC, a command‐line‐based tool that takes FASTQ/FASTA files as input and performs QC [39]. The data (FASTQ) are then forwarded to the Trimmomatic tool [40, 41] for the trimming of low‐quality sequences, filtering adapters, and base cutting. The output of the Trimmomatic is processed FASTQ. The Burrows–Wheeler Aligner (BWA) tool is then used for mapping sequence data against the reference human genome (hg38) [42, 43, 44]. BWA produces a SAM file, which is then passed to the SortSam [45] tool for sorting by the reference sequence name and left‐most mapping position. The sorted SAM file is then inputted to the MarkDuplicates [46] to locate, tag, and remove duplicate reads, and enhance the quality of the alignment and reduce the number of variant false positives. The outcome of the MarkDuplicates is again sorted and indexed file. Next, the index tool from SAMtools is used to create and index file, allowing to access faster.

The JWES implements the GATK [19, 47, 48] to find SNPs and INDELs. The GATK locally realign reads to reduce the number of false positives. This has been performed in two separate steps: (a) determine intervals with RealignerTargetCreator and (b) realign intervals with IndelRealigner. Next, BaseRecalibrator is used to adjust the quality scores by using an empirical machine learning (ML) model. The output of both BaseRecalibrator steps is two separate recalibration tables. AnalyzeCovariates inputs these two recalibrations tables and produces multiple lines and bar plots that show the quality of the recalibration. Next, HaplotypeCaller is used to call SNPs and INDELs, which produces a file that contains all the variants found. The rest of the operations in this pipeline are performed twice and parallel, once for the SNPs and once for the INDELs. SelectVariants is used to separate the SNPs from the INDELs, which results two variant files variant call format (VCF). The JWES applies VariantRecalibrator to create a Gaussian mixture model and produce high‐quality cluster of SNPs. It is done by looking at the distribution of values over the input call set, and then, the scoring is reassigned to each of the variants. ApplyRecalibration is followed, which applies a score cutoff, and filters out variants that fall below the specified sensitivity threshold. SelectVariants is then used to select the variants that have successfully passed the ApplyRecalibration step. The following step in the pipeline is to calculate the QC metrics of the variants with VariantEval. These metrics include the total number of SNPs and the ratio of transition variants to transversions. The output is a table that details all the metrics described above for each of the variants. VariantsToTable is then used to extract the fields from the variants and transforms it into a table. The JWES computes histograms, per‐base reports, and coverage for a given genome using the genomecov, and uses the snpEff to annotate and predict the effects of the variants by using an interval forest approach. The outcome of the JWES is an annotated variant file.

**Fig. 2:**
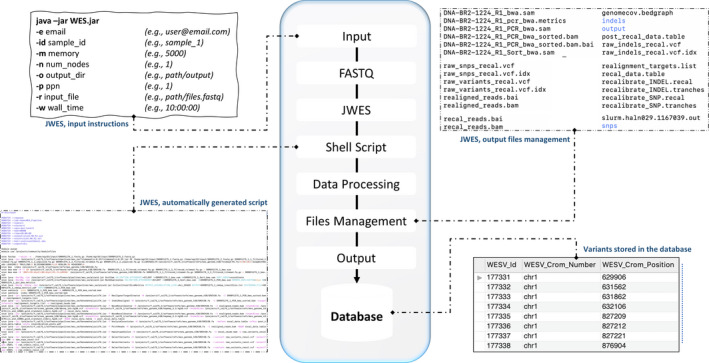
JWES pipeline data and workflow. The figure explains overall roadmap of JWES, which includes input preparation, automatics script generation, output files management, and variants data storage in database

To customize the JWES, it is required to modify the configuration file, which contains all the paths to the bioinformatics applications used within pipeline (Fig. 2). Once the configuration file will be modified, the users only need to call the JWES and provide the login information of the HPC cluster (only if applicable, optional), project name, path, or paths to all the sample files (FASTQ), and number of nodes that will be used to deploy the computation. At successful execution, the JWES will automatically generate, load, and execute the script into the HPC cluster. Once completed, the variants file produced in the snpEff step will be parsed and automatically uploaded into the connected MySQL database management server for later downstream analysis and visualization. We have programmed the JWES in Java‐8 using Eclipse IDE 2020‐09 and have successfully tested using Microsoft Windows 10, macOS Big Sur (version 11.2.2), and UNIX operating systems. Its functionality has been validated and reproduced multiple times within the HPC environment (deployed with Slurm Workload Manager) provided by the Rutgers University.

### JWES variant data storage

**Fig. 3:**
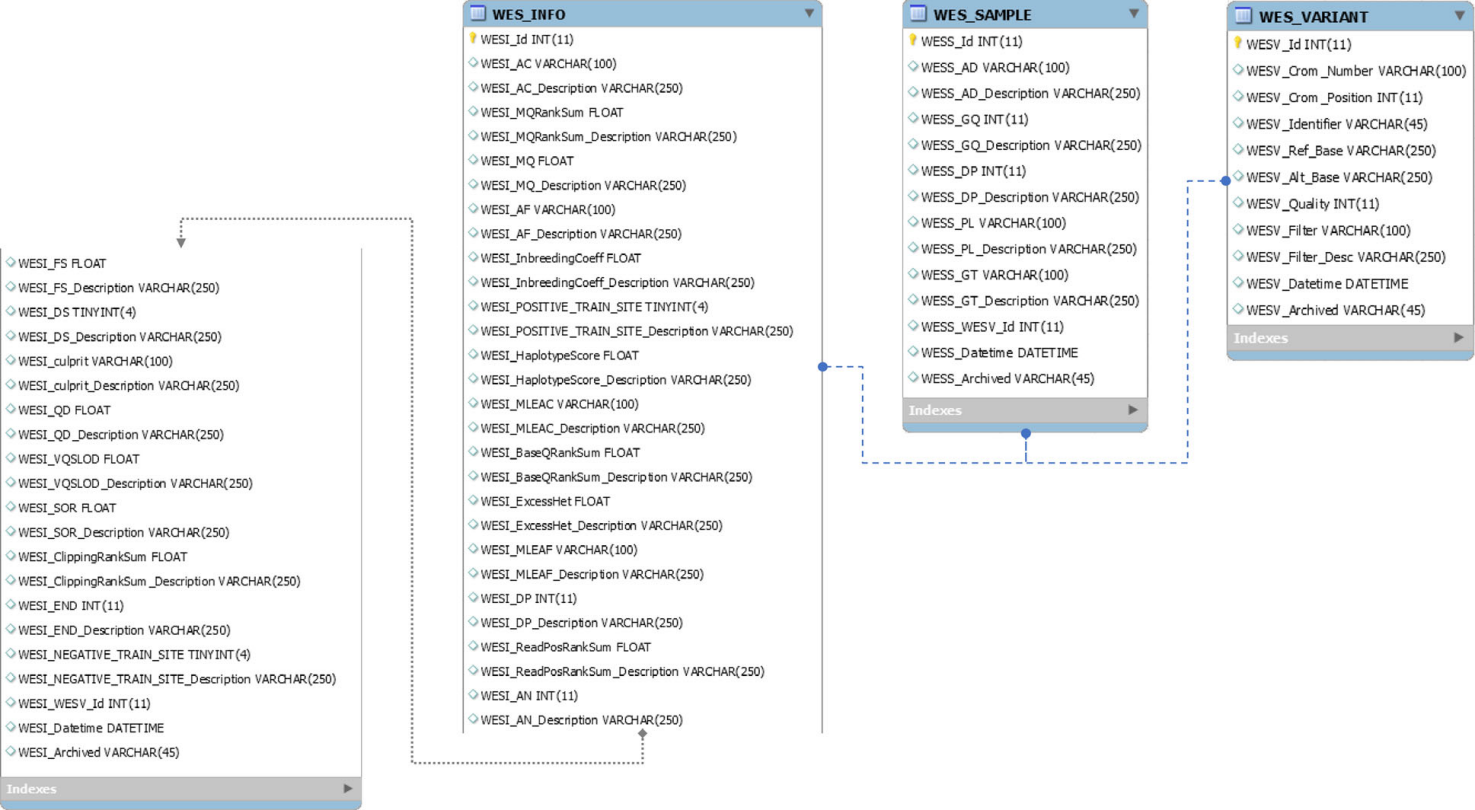
JWES database design. The figure explains ERD of JWES database, which includes three tables: WES Info, WES Samples, and WES Variant

The JWES implements data storage module to overcome the limitation of efficiently managing high‐volume gene‐variant data management and timely support downstream analysis, interpretation, and presentation. The JWES data storage module consists of two parts: (a) modeled relational database, and (b) java‐based application for efficient data extraction, transfer, and loading (ETL) from source variant (CSV) files to connected database server, that is, JWES‐ETL. Designed entity relationship diagram (ERD) of implemented database is organized into three separate tables/relations: (a) Variant, (b) Info, and (c) Sample (Fig. 3). Variant table contains the most important information (e.g., chromosome, reference position, reference base, alternate base, filters, filters passed, data quality, mapping quality, genotype, genotype quality, allele count, combined depth, record if somatic mutation, allelic depths, and phred) extracted from the variant files (CSV), produced by the snpEff. Info table contains additional information that has been reported with discovered variants, and sample stores genotype information.

We have developed the JWES‐ETL module as a separate cross‐platform application. However, the JWES embeds it in pipeline, as one of the final steps. The JWES‐ETL module extract variants from the VCF file. Extracted data are then cleansed, classified, reformatted, and uploaded into the database server. The input instructions of the JWES‐ETL module are simple and consist of username, password, and connection details of available database server, where variant data will be stored. Database server can be deployed locally and an independent network‐based server. In any case, it should be able to access and get data streamed through HPC environment, where the JWES will deploy and execute pipelines. The reason to develop the JWES‐ETL as an independent module is to support bioinformaticians who have already developed their variant calling pipelines but cannot efficiently perform automated variant data management using database management system.

### JWES variant data visualization

Data visualization is considered essential for the variant data interpretation, as it bridges the gap between algorithmic approaches and the cognitive skills of users and investigators. Over the past decade, different visualization tools have been emerged to display data in different categories including but not limited to dot plot, scatter plot, circos, two‐way view, linear coordinate plot, multiway view, graph view, linear genome browser, SV table, and population view [49]. Each of these visualization kinds is important and significant. However, in this study we are interested in implementing the one that is appropriate for showing variable genome features including distribution of variant data, variation in genome structure, inter‐chromosomal variants, positional relationships between genomic intervals, genomic rearrangements, and producing ideograms in any order and orientation. Based on the requirements and best open‐source application programming interface (API) availabilities, we choose to implement circos graphs [50].

We developed the JWES data visualization module for plotting the circos graphs [50] based on the variants stored in the database, using the JWES‐ETL module. The JWES data visualization module is another cross‐platform application programmed in Python programming language. It queries (SQL) variants from the database and plots circos graphs by iterating through each of the variants. Data visualization process starts by creating a list of unique genes, then counting total number of variants for each, and using the Ensembl API to find chromosome number, start and end positions of genes and variants. The output of the JWES visualization module is a text formatted file that can be directly passed to circos tool to plot the graphs. Likewise, the JWES‐ETL module, it can be embedded as part of the JWES pipeline and executed as a standalone application (without using HPC environment) as well. JWES users’ guidelines are provided in the attached Supplementary Material.

## Results

The performance of the JWES has been tested and validated in‐house at different experiments. We have successfully applied the JWES for the processing, management, and gene‐variant discovery, annotation, prediction, and genotyping of the WGS and WES data sequenced for analyzing variable complex disorders (e.g., Alzheimer, Arthritis, Asthma, Diabetes mellitus, Heart failure, Hypertension, Obesity, Osteoporosis, and multiple Cancer disorders). Overall, we have produced an in‐house database of over 1000 million SNPs using the JWES.

### Variant calling with JWES

In this manuscript, we report performance of the JWES with some reproducible case studies, using open access (Table 1) [51, 52, 53, 54, 55] and in‐house generated, high‐quality datasets (Table 2). We created a cohort of publicly available data, which included total 14 whole genome sequenced samples (Table 1). The JWES pipeline delivered promising results by offering a wider range of variants (4 803 792). First, we applied the JWES to the WGS sample (SRR12474733) of project PRJNA657985, which resulted in 43 685 variants [52]. This sample was collected in Sweden and from the patient of severe acute respiratory syndrome coronavirus 2 (SARS‐CoV‐2). Next, we applied the JWES to some other WGS samples (SARS‐CoV‐2) from projects including, PRJNA657985 (SRR12486921), PRJNA657938 (SRR12486921), PRJNA624223 (SRR12328890), PRJEB39632 (ERR4387385, ERR4387386, ERR4387388), PRJNA649101 (SRR12336742, SRR12336753, SRR12336755, SRR12336756, SRR12336761, SRR12336765, SRR12336766), and PRJNA207663 (SRR891275) [53, 54, 55]. The JWES reported total 2 736 453 variants for PRJNA657938, 1 793 959 variants for PRJNA624223, 154 016 variants for PRJEB39632, 54 930 variants for PRJNA649101, and 20 749 variants for PRJNA207663. Results are reported in Table 1, including data type, project ids, sample numbers and ids, total variant count, sources URL, and date last accessed.

**Table 1:** List of publicly available NGS datasets and extracted total number of variants using JWES. Table 1 provides an overview of different whole genome/exome sequencing projects selected for variant discovery using JWES. Table 1 includes data type, project ids, sample numbers and ids, total variant count, sources URL, and date last accessed.

Data Type	Project IDs	Sample numbers	Sample IDs	Total variants	Source URL	Date accessed
WGS	PRJNA657985	1	SRR12474733	43 685	https://www.ebi.ac.uk/ena/browser/view/PRJEB39632	06‐28‐2021
WGS	PRJNA657938	1	SRR12486921	2 736 453	https://www.ebi.ac.uk/ena/browser/view/PRJNA657985	06‐28‐2021
WGS	PRJNA624223	1	SRR12328890	1 793 959	https://www.ebi.ac.uk/ena/browser/view/PRJNA657938	06‐28‐2021
WGS	PRJEB39632	3	ERR4387385, ERR4387386, ERR4387388	154 016	https://www.ebi.ac.uk/ena/browser/view/PRJNA649101	06‐28‐2021
WGS	PRJNA649101	7	SRR12336742, SRR12336753, SRR12336755, SRR12336756, SRR12336761, SRR12336765, SRR12336766	54 930	https://www.ebi.ac.uk/ena/browser/view/PRJNA624223	06‐28‐2021
WGS/ATAC‐seq	PRJNA207663	1	SRR891275	20 749	https://trace.ncbi.nlm.nih.gov/Traces/sra/?run=SRR891275	02‐28‐2021

**Table 2:** JWES performance evaluation details based on processed high‐quality—in‐house generated WGS datasets. Table 2 provides an overview of processing time (hours) taken by the JWES pipeline to complete the task of variant calling. The performance of the JWES is based upon number of features including, the size of sample (RAW Data), VCF file size, Memory, Nodes, and designated CPUs‐per‐task.

Sample IDs	Total variants	RAW data – sample sizes	VCF file size (SNP and Indel)	Time (h)	Number of nodes	CPUs – per – tasks	Memory
1	4 867 674	1.2 TB	2.6 GB 654 MB	65	1	8	46G
2	4 928 789	1.5 TB	2.6 GB 678 MB	74	1	8	46G
3	5 808 057	1.7 TB	3.1 GB 812 MB	77	1	8	46G
4	4 897 749	1.3 TB	2.6 GB 657 MB	61	1	8	46G
5	4 883 410	1.4 TB	2.6 GB 671 MB	70	1	8	46G
6	4 983 681	1.6 TB	2.6 GB 698 MB	83	1	8	46G
7	5 000 735	1.5 TB	2.6 GB 698 MB	88	1	8	46G
8	5 902 241	1.9 TB	3.1 GB 837 MB	95	1	8	46G
9	4 870 099	1.1 TB	2.6 GB 654 MB	57	1	8	46G
10	4 925 968	1.3 TB	2.6 GB 675 MB	67	1	8	46G

**Fig. 4:**
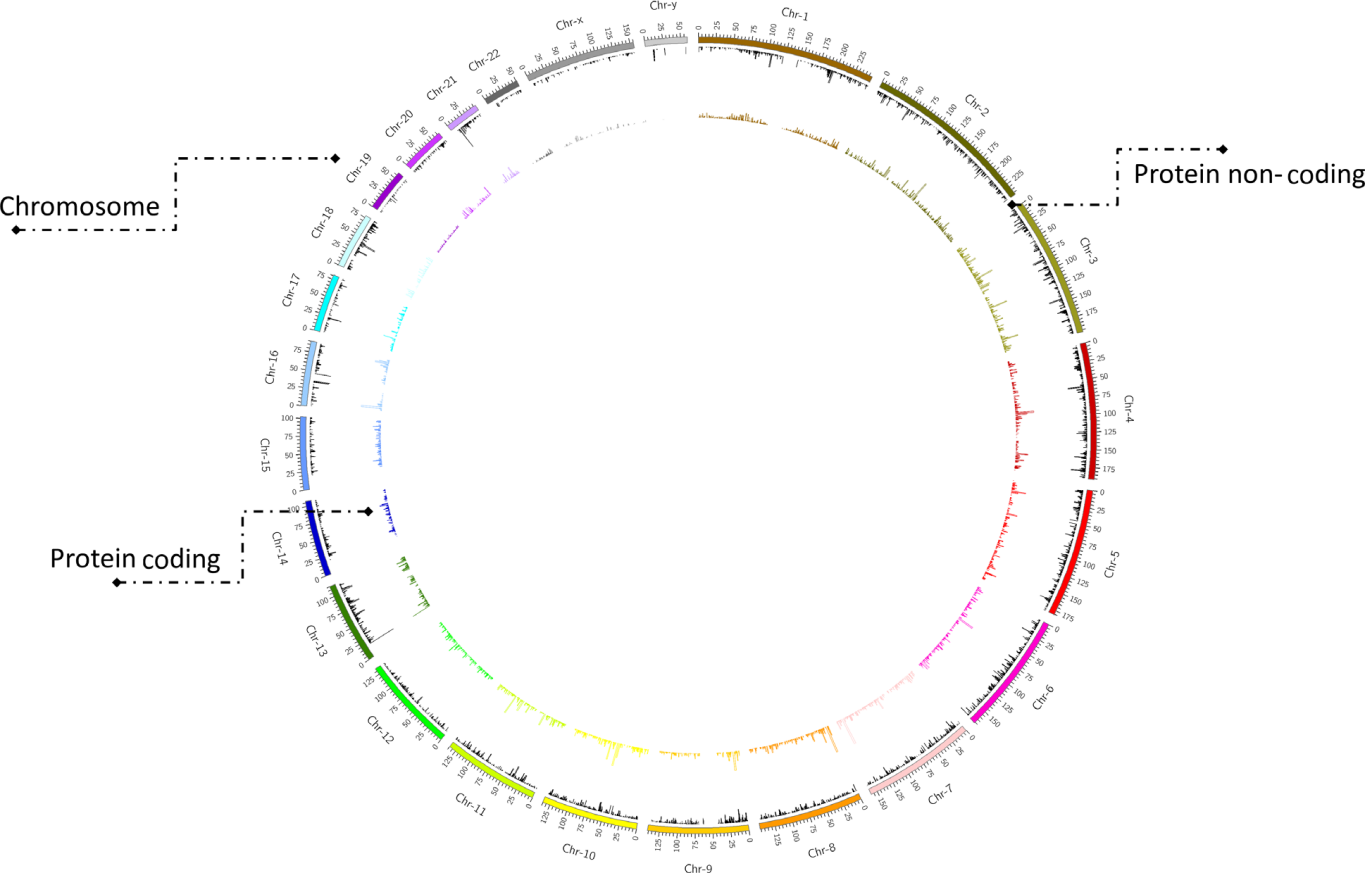
JWES visualization. The figure presents Circos graph plotting all the variants for all chromosomes. The internal histogram represents the total number of variants found in the protein‐coding genes, and the external histogram represents variants found in the noncoding genes

To further test the JWES pipeline, we downloaded SRR891275 from NCBI Sequence Read Architecture (SRA) database. The SRR891275 was submitted by the Gene Expression Omnibus (GEO). The sequencing dataset was derived from purified CD4+ T cells from human samples (Homo sapiens) using Illumina HiSeq 2000 [51]. We applied the JWES pipeline at the SRR891275 within HPC environment, provided by the Rutgers Office of Advanced Research Computing (OARC). We were able to successfully complete the execution of deployed pipeline and using the JWES‐ETL module, all the variants were successfully extracted from the CSV file and stored in the relational database management server, provided by the Rutgers Institute for Health (IFH). We identified a total of 20 749 variants across all chromosomes. Then, mapped each variant to the gene that it has affected and divided the variants into two groups: variants that affect protein‐coding genes and noncoding genes. We were able to discover a total of 12 979 variants (˜ 63%) that affected noncoding genes, and a total of 7770 variants (˜ 37%) that affected protein‐coding genes. We found 12 979 noncoding genes and the 7770 protein‐coding genes. We observed that ˜ 0.8% of the noncoding genes have > 10 variants, and the remaining ˜ 99.2% have ≤ 9 variants. Similarly, for the protein‐coding region only ˜ 2% of those genes have ≥ 10 variants and remaining ˜ 98% have ≤ 9 variants. Next, we reported chromosome‐based variant distribution in a simplified circos plot, divided in two histograms, (a) color‐coded and black (Fig. 4). Color‐coded histogram presents all the variants that appeared in protein‐coding genes, and the black in noncoding genes. We observed protein‐coding region of chromosome‐2 including ˜ 7.4% of the total variants, being the chromosome with the highest number of variants in the protein‐coding region. Similarly, chromosome‐2 reported ˜ 11.8% of the total variants for the noncoding region, the highest among all chromosomes in noncoding regions. Variant spectrum of chromosome‐Y contains only ˜ 0.5% of the variants for the noncoding region, being the chromosome with the smallest number of noncoding variants. Similarly, chromosome‐Y only had ˜ 0.03% of variants in the protein‐coding section, being the chromosome with the lowest number of variants in the protein‐coding region.

### Performance evaluation of JWES

In this manuscript, we report evaluated performance of the JWES by testing it at our in‐house generated and processed HQ WGS samples. We submitted 10 parallel jobs within HPC environment, supported by the Rutgers OARC. These jobs were scripted and deployed by the JWES with Slurm, and for 10 different WGS samples, sized (raw, FASTQ) between 1.2 and 1.9 TB (Table 2). Individual job configurations include requesting 1 dedicated node with 8 CPUs‐per‐task and 46G Memory. Overall data processing took time in between 61 and 95 h, and the final VCF files produced—ranged between 2.6 GB and 3.1 GB of size including over 4 million variants resulted for each pipeline. We would like to emphasize the fact that data processing speed varies based upon the computing infrastructure.

## Discussion

We emphasize that automated data processing, management, and visualization should be an indispensable component of the modern WGS and WES data analysis, which is currently not the case. The processed high‐quality WGS and WES data (e.g., generated by Illumina HiSeq) concludes with, if not millions then over hundred thousand variants. Downstream analysis of dataset including few samples can be well managed by the small team of bioinformaticians. However, investigating susceptibility of multiple samples (e.g., hundred/thousands) is cumbersome, tedious, and time‐consuming. It is still a challenging task today to perform automatic downstream analysis, which includes gene‐variant discovery, annotation, prediction, and genotyping. Furthermore, it is difficult to timely detect De Novo Single‐Nucleotide Variants (DNSNVs) [56] and minimize the number of false negatives [57]. Implementing platforms dealing big data analytic challenges require manpower (e.g., bioinformaticians, biostatisticians), computational resources (e.g., HPC and cloud computing environments), and bioinformatics applications (e.g., data inspection, mapping to reference genomes, expression analysis, and variant calling).

The exome sequencing has enabled us to specifically target and sequence the protein‐coding region of the hg38. In the last several years, it has served as a powerful and cost‐effective tool for minutely dissect the genetic basis of diseases including the Mendelian disorders [58]. It has catapulted the speed of novel disease‐associated genes identification in intellectual disability [59], Parkinson's disease [60], and cancers [61]. Data generated from NGS technologies have led to a paradigm shift in the field of medical research and how clinical investigators practice the treatment of rare and more frequent human disorders [62]. It has always been one of the fundamental pursuit of genetics to determine genotype–phenotype association. The state of art sequencing technologies not only provide enormous genome‐wide data but are also inexpensive compared to previous times [63]. However, one of the biggest challenges is to develop constantly evolving, analytical bioinformatic pipelines that can process and analyze data to identify the variants [64]. The wealth of existing bioinformatics tools, databases, and applications have helped in accelerating the data processing and analysis, and however, there is a lacunae of simpler clinician/patient friendly tools/pipelines for a broader utility in clinical personalized medicine setting [65, 66].

With the development of the JWES, the expectation is to aid the identification process of disease associated, clinically relevant variants in a patient/individual's genome. To summarize, in this manuscript, we have presented the JWES; a new bioinformatics pipeline for gene‐variant discovery, annotation, prediction, and genotyping. While the JWES development, we were focused on testing the performance of most used bioinformatics applications for the WGS/WES data processing and analysis, addressing big data analytics and application usability issues. The JWES is a user‐friendly application, which can be easily configured and used by the noncomputational and bench scientist. It implements an efficient data ETL process to store identified variants in a database. The JWES database features include but are not limited to the management of variant information about genomic position, reference and alternate base of variants, chromosome, alleles, filters, and genotype quality. Furthermore, it offers data visualization module with several features to automatically generate circos plots, which facilitate interactive graphing of gene‐variant data. Circos features plotting of complex data in multiple tracks of different types (histograms, scatterplot, highlights, tiles, text), configurations (ticks, rules), color schemes, customized ideogram, and networked connections dependent on their values [67, 68]. The JWES visualization module enables designing of readable outlines and reducing variant analysis pipeline outputs to produce a summarized image with digestible fashion.

The growing focus toward the personalized medicine approach is ushered by a fundamental shift in one size fits all, to a precisely more specific treatment plan for patients keeping predisposing factors/ conditions in mind. The precision medicine approach requires state of art development in the healthcare technologies that can foster the successful incorporation of heterogeneous genomic data into clinical settings. Currently, there is a lack of genomics pipelines that can efficiently process and analyze genomic data (WGS/WES). With the development of the JWES, we hope to help the clinical and scientific communities in moving a step closer toward the precision medicine approach with a more standardized and consistent way. In the clinical settings, the outcome of the JWES pipeline can be used for the predictive analysis, and deep phenotyping by integrating the processed variant data in an AI/ML ready formats.

## Conclusions

The scope of this study is to support the process of genetic testing with the classification of susceptibility genes to detect changes of clinical significance. We have presented and validated the JWES to deal with handling high‐volume variant data management, analysis, and visualization challenges. The current version of the JWES offers command line but an easy‐to‐use interface. However, in future we are looking forward to implementing interactive interface, which will further ease the job for its users to process and analyze the WGS and WES data. Furthermore, we will be advancing data visualization module to produce dynamic heat maps, gene pathways and networks, and cluster maps for gene expression and variant analysis.

## Conflict of interest

The authors declare no conflict of interest.

## Author contributions

ZA proposed and lead the study. SZ and ZA designed, and EGR programmed JWES. ZA, SZ, DM, and EGR validated JWES and supported this study. ZA drafted, and all authors participated in writing and review of the manuscript. All authors have approved the manuscript.

## Supporting information

Supplementary Material JWES installation and configuration instructions.

## Data Availability

The data that support the findings of this study are openly available, and details are provided in Table 1. The source code of the JWES, and data that supports the findings of this study are freely available through GitHub: JWES database <https://github.com/drzeeshanahmed/JWES‐DB>, JWES variant <https://github.com/drzeeshanahmed/JWES‐Variant>, and JWES visualization module <https://github.com/drzeeshanahmed/JWES‐Visual>.
