# Supplementary material for: JWES: a new pipeline for whole genome/exome sequence data processing, management, and gene‐variant discovery, annotation, prediction, and genotyping
Source: FEBS Open Bio. 2021 Aug 11;11(9):2441–52. doi: 10.1002/2211-5463.13261 (PMC8409305; doi:10.1002/2211-5463.13261)
Supplement: Supplementary file 1 — Supplementary Material JWES installation and configuration instructions. [file FEB4-11-2441-s001.pdf]

Supplementary Material 1:

JWES guidelines, installation, and configuration instructions.

Title:

JWES: A new pipeline for whole genome/exome sequence data processing, management, and gene-variant discovery, annotation, prediction, and genotyping

Authors:

Zeeshan Ahmed[1, 2, *], Eduard Gibert Renart[1], Deepshikha Mishra[1], Saman Zeeshan[3]

Affiliations:

1. Institute for Health, Health Care Policy and Aging Research, Rutgers, The State University of New Jersey, 112 Paterson Street, New Brunswick, NJ, USA.
2. Department of Medicine, Rutgers Robert Wood Johnson Medical School, Rutgers Biomedical and Health Sciences, 125 Paterson Street, New Brunswick, NJ, USA.
3. Rutgers Cancer Institute of New Jersey, Rutgers, The State University of New Jersey, 195 Little Albany St, New Brunswick, NJ, USA.

***Corresponding author:**

Zeeshan Ahmed, Institute for Health, Health Care Policy and Aging Research, Rutgers, The State University of New Jersey, 112 Paterson Street, New Brunswick, NJ, USA.

Email: zahmed@ifh.rutgers.edu

GVViZ © Ahmed Lab, Rutgers University, 2021.

Table of Contents

1. Compilation of JWES Tools 3

1.1 Eclipse IDE Compilation........ 3

2.2 Command Line Compilation 4

2. Configuration of JWES Pipeline........ 5

3. Installation of JWES Pipeline 6

4. Installation and Configuration of JWES VCF Parser........ 6

5. Installation and Configuration of JWES Visualization Tool 6

6. Declarations 7

6.1 Ethical Approval and Consent to participate 7

6.2 Consent for publication........ 7

6.3 Availability of data and material 7

6.4 Competing interests........ 7

6.5 Funding........ 7

7. Acknowledgements........ 7

1. Compilation of JWES Tools

To compile all the Java tools that JWES requires Apache Maven to be installed (https://maven.apache.org/).

There are two options to compile all the JWES Tools: 1) Using Eclipse IDE 2) Using Command Line.

1.1 Eclipse IDE Compilation

Import the JWES Project into Eclipse by File -> Import.

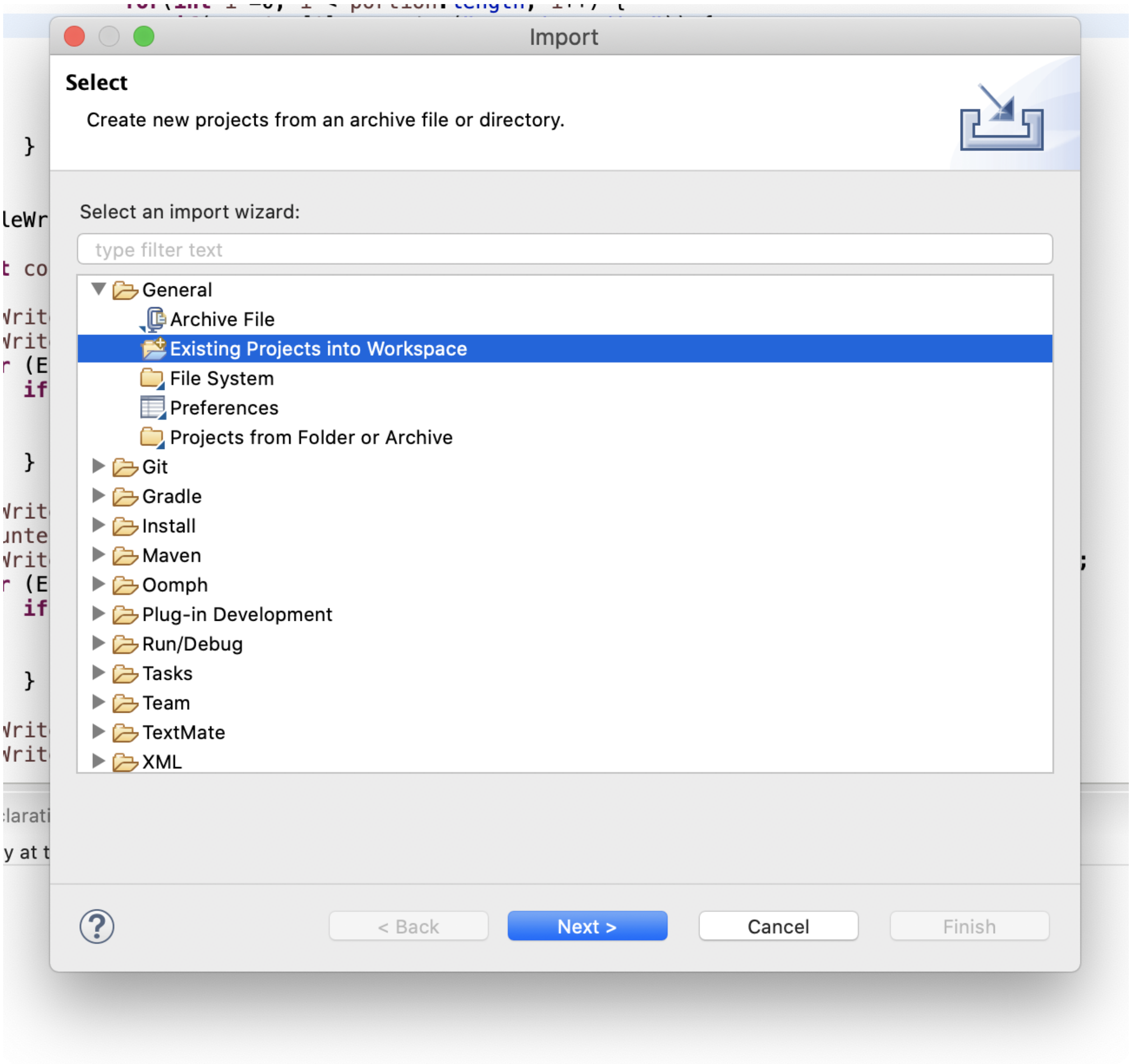

Then select General -> Existing Projects into Workspace, click Next. Then click browse... and find the location of where JWES Tool is located and click Finish.

Once the project has been successfully imported. Then right click on the project name and click on Run as -> Maven Build…

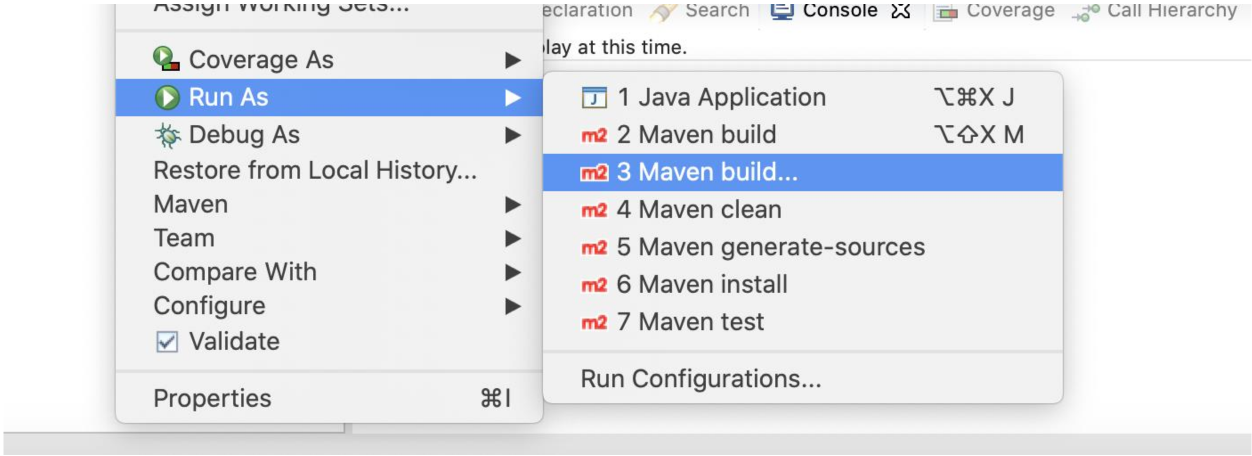

A window will pop up, look for a text box with the name of **Goals:** in the text box type **package**.

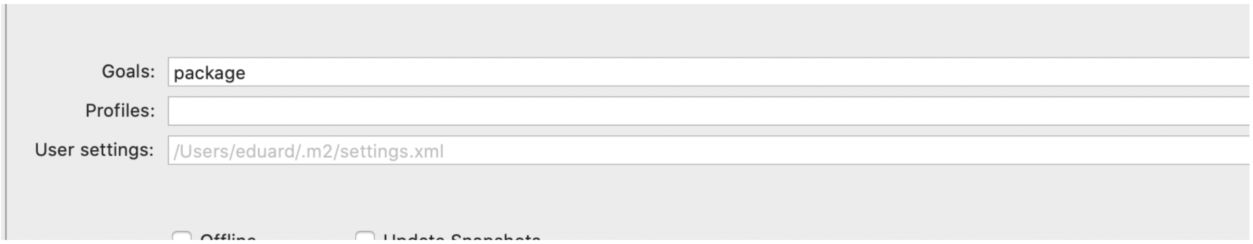

Click Apply and then Run. The JWES Tool has been successfully compiled. The JAR file is located in the Root folder under the **Target** folder.

2.2 Command Line Compilation

Open a command line and "cd" into the Root directory of the JWES Tool. Once there, type in the command line:

mvn clean package

Once completed a **Target** folder containing all the jar files will appear in the root directory. To run it use the file with the name **JWES-TOOL-NAME-jar-with-dependencies.jar.**

2. Configuration of JWES Pipeline

Before JWES Pipeline can be deployed into the HPC cluster, there is a need to configure and make sure all the required applications are installed properly in the HPC environment. Navigate to the JWES Pipeline root directory then **src/main/resources.** There is a file called: **config.properties**, open it with a text editor:

```
#WES Pipeline Properties
Job.Name=JOB_NAME
Cluster.Host.Name=HOST_NAME

Hg38.Path=/PHATH/GRCh38.fa
Dbsnp.Path=/PHATH/dbsnp_146.hg38.vcf
Hapmap.Path=/PHATH/hapmap_3.3.hg38.vcf
Mills.Path=/PHATH/Mills_and_1000G_gold_standard.indels.hg38.vcf
Omni.Path=/PHATH/wes_genome_h38/1000G_omni2.5.hg38.vcf
Phase1.Path=/PHATH/1000G_phase1.snps.high_confidence.hg38.vcf
TruSeq3.Path=/PHATH/TrueSeq3

ModuleLoad.Fastqc=False
ModulePath.Fastqc=/PHATH/fastqc_v0.11.9-yc759

ModuleLoad.Trimmomatic=False
ModulePath.Trimmomatic=/PHATH/trimmomatic-0.39.jar

ModuleLoad.Picard=False
ModulePath.Picard=/PHATH/picard.jars

ModuleLoad.Bwa=False
ModulePath.Bwa=/PHATH/bwa-0.7.17-yc759

ModuleLoad.Samtools=True
ModulePath.Samtools=/PHATH/samtools/

#Needs to be gtk3
ModuleLoad.Gatk=False
ModulePath.Gatk=/PHATH/GenomeAnalysisTK.jar

ModuleLoad.SnpEff=False
```

Edit the file accordingly to make sure that all the paths for all the software are installed and inputted correctly. Used version for the JWES pipeline and other detail are given in S-Table 1.

No.	Application	Version	Download Web Link
1	FASTQC	0.11.9	https://www.bioinformatics.babraham.ac.uk/projects/fastqc/
2	Trimmomatic	0.39	
3	Burrows-Wheeler Alignment	0.7.17	http://www.usadellab.org/cms/?page=trimmomatic
4	Picard	2.23.6	https://github.com/broadinstitute/picard
5	SamTools	1.8	http://www.htslib.org/
6	GATK	3.8	https://gatk.broadinstitute.org/hc/en-us
7	SnpEff	5.0	https://pcingola.github.io/SnpEff/
8	BedTools	2.25.0	https://bedtools.readthedocs.io/en/latest/

S-Table 1 – Software requirement need it, in order to successfully run JWES.

3. Installation of JWES Pipeline

The software executable (JAR file) is open source and freely available, in order to execute JWES, the only requirements is the installation of Java Runtime Environment and have installed in **(https://www.oracle.com/java/technologies/javase-jre8-downloads.html)**

Before executing JWES, it is important to set valid applications paths and calling protocols (section: Configuration of JWES Pipeline).

To run the program do as following:

Java -jar VCF.jar *127.0.0.1 3306 username password vcf_to_upload.vcf*

4. Installation and Configuration of JWES VCF Parser

The software executable (JAR file) is open source and freely available and to execute the JWES VCF Parser, the only requirement is the installation of Java Runtime Environment (https://www.oracle.com/java/technologies/javase-jre8-downloads.html)

To run the JWES VCF Parser we only need to pass a few command line arguments:

1. Database IP Address ex: 127.0.0.1
2. Database port, if using MySQL, the default port is 3306.
3. Username of the database.
4. Password for the database, blank if no password.
5. VCF file to upload to the DB.

To run the program do as following:

Java -jar VCF.jar *127.0.0.1 3306 username password vcf_to_upload.vcf*

5. Installation and Configuration of JWES Visualization Tool

The software executable (Python file) is open source and freely available and to execute the JWES Visualization Tool, the only requirement is the installation of Python 2.x (https://www.python.org/downloads/) and the following python modules:

1. ensembl-rest 0.3.3 (https://pypi.org/project/ensembl-rest/)
2. requests 2.24.0 (https://pypi.org/project/requests/)

To run the JWES Visualization Tool we only need to pass the name of the CSV file that we have downloaded from the previous step. To run the program do as following:

python Graph.py *file_name_to_process.csv*

The output of the program is two sperate **.txt** files Histogram.txt which will contain all the information formatted and ready for Circos Tool to plot, and a Histogram_Missing.txt which will contain any of the genes that has not found.

6. Declarations

6.1 Ethical Approval and Consent to participate

Not applicable

6.2 Consent for publication

Not applicable

6.3 Availability of data and material

The source code of JWES, and data that supports the findings of this study are freely available through GitHub: JWES database <https://github.com/drzeeshanahmed/JWES-DB>, JWES variant <https://github.com/drzeeshanahmed/JWES-Variant>, and JWES visualization module <https://github.com/drzeeshanahmed/JWES-Visual>.

6.4 Competing interests

The authors declare that they have no competing interests.

6.5 Funding

This work was supported by the Institute for Health, Health Care Policy and Aging Research, and Robert Wood Johnson Medical School, at Rutgers, The State University of New Jersey.

7. Acknowledgements

We appreciate great support by the Rutgers Institute for Health, Health Care Policy and Aging Research (IFH); Department of Medicine, Rutgers Robert Wood Johnson Medical School (RWJMS); and Rutgers Biomedical and Health Sciences (RBHS), at the Rutgers, The State University of New Jersey.

We thank students, members, and collaborators of the Ahmed Lab at the Rutgers IFH and RWJMS for their active participation and contribution to this study.

We are grateful for research services and resources provided by the Institute for Health Survey/Data Core at Rutgers University. We especially thank to Mr. Dinesh Mendhe, Mr. Jonathan Thompson, and Mr. Sam Mirmajlesi for their support.

We acknowledge the Office of Advanced Research Computing (OARC) at Rutgers, The State University of New Jersey for providing access to the Amarel cluster and associated research computing resources that have contributed to the results reported here.

GVViZ © Ahmed Lab, Rutgers University, 2021.
